# Factors Associated with Adherence to Anti-Hypertensive Treatment in Pakistan

**DOI:** 10.1371/journal.pone.0000280

**Published:** 2007-03-14

**Authors:** Saman K. Hashmi, Maria B. Afridi, Kanza Abbas, Rubina A. Sajwani, Danish Saleheen, Philippe M. Frossard, Mohammad Ishaq, Aisha Ambreen, Usman Ahmad

**Affiliations:** 1 Medical College, Aga Khan University, Karachi, Pakistan; 2 Department of Biological and Biomedical Sciences, Aga Khan University, Karachi, Pakistan; 3 National Institute of Cardiovascular Diseases, Karachi, Pakistan; 4 Department of Family Medicine, Aga Khan University, Karachi, Pakistan; James Cook University, Australia

## Abstract

**Objectives:**

Poor adherence is one of the biggest obstacles in therapeutic control of high blood pressure. The objectives of this study were (i) to measure adherence to antihypertensive therapy in a representative sample of the hypertensive Pakistani population and (ii) to investigate the factors associated with adherence in the studied population.

**Methods and Results:**

A cross-sectional study was conducted on a simple random sample of 460 patients at the Aga Khan University Hospital (AKUH) and National Institute of Cardiovascular Diseases, Karachi, from September 2005–May 2006. Adherence was assessed using the Morisky Medication Adherence Scale (MMAS), with scores ranging from 0 (non-adherent) to 4 (adherent). In addition to MMAS, patient self-reports about the number of pills taken over a prescribed period were used to estimate adherence as a percentage. AKU Anxiety and Depression Scale (AKU-ADS) was incorporated to find any association between depression and adherence. At a cut-off value of 80%, 77% of the cases were adherent. Upon univariate analyses, increasing age, better awareness and increasing number of pills prescribed significantly improved adherence, while depression showed no association. Significant associations, upon multivariate analyses, included number of drugs that a patient was taking (*P*<0.02) and whether he/she was taking medication regularly or only for symptomatic relief (*P*<0.00001).

**Conclusions:**

Similar to what has been reported worldwide, younger age, poor awareness, and symptomatic treatment adversely affected adherence to antihypertensive medication in our population. In contrast, monotherapy reduced adherence, whereas psychosocial factors such as depression showed no association. These findings may be used to identify the subset of population at risk of low adherence who should be targeted for interventions to achieve better blood pressure control and hence prevent complications.

## Introduction

Hypertension is an overwhelming global challenge which ranks third as a cause of disability-adjusted life-years [1]. According to the Seventh Report of the Joint National Committee on Hypertension, there are approximately 50 million hypertensive individuals in the United States only and 1 billion worldwide [2]. Even though the burden of hypertension is currently centered in economically developed countries (37.3%), developing countries will feel a greater impact due to their larger population proportion. Indeed estimates indicate that up to three-quarters of the world's hypertensive population will be in economically developing countries by the year 2025 [1].

The National Health Survey of Pakistan (NHSP), conducted from 1990 to 1994, showed that hypertension affects 18% of adolescents above 15 years of age and 33% of adults above 45 years of age; less than 3% hypertensive patients, however, have their BP controlled to 140/90 mm Hg or below and more than 70% of all hypertensive patients (85% in rural areas) in Pakistan are not even aware of their disease [3].

Studies worldwide indicate that despite the availability of effective medical therapy, over half of all hypertensives do not take any treatment [4] and more than half of those on treatment have blood pressures over the 140/90 mmHg threshold [5]. The World Health Organization (WHO) describes poor adherence as the most important cause of uncontrolled blood pressure and estimates that 50–70% of people do not take their antihypertensive medication as prescribed [6].

It has been well documented that uncontrolled blood pressure increases the risk of ischemic heart disease 3-to 4-fold [7] and the overall cardiovascular risk by 2-to 3-fold [8]. The incidence of stroke increases approximately 3-fold in patients with borderline hypertension and approximately 8-fold in those with definite hypertension [9]. Moreover, a recent case control study has shown that non adherence to therapy is associated with an increased risk of stroke in patients with hypertension [10], [11].

Adherence is defined by WHO as “the extent to which a person's behavior–taking medication, following a diet, and/or executing lifestyle changes-corresponds with agreed recommendations from a health care provider” [12]. Adherence is dependent on numerous factors and has been shown to vary from 0 to 100% in different populations studied [12], [13], [14], [15]. Factors such as age [16], [17], gender [18], low socioeconomic status and severity of disease [17], class of drug prescribed [19], number of pills per day [16], [18], side effects of medication [16], [17], patient's inadequate understanding of the disease and importance of the treatment [16], [17], co-morbid medical conditions [17], lack of social support [20], poor patient-provider relationship [21], cost, forgetfulness [22], and presence of psychological problems, especially depression [17], [21], have all been shown to affect adherence in various populations.

We undertook this research with the objectives of (i) measuring adherence through the use of validated tools and (ii) investigating the demographic, social and personal factors affecting patient adherence to antihypertensive therapy in the Pakistani population.

## Methods

### Study design and sample recruitment

This descriptive study was a questionnaire-based cross sectional analysis. A simple random sample of 460 patients was selected between September 2005 and May 2006 from two tertiary care hospitals in Karachi, Pakistan, namely Aga Khan University Hospital (AKUH) and National Institute of Cardiovascular Diseases (NICVD).

AKUH is one of the most advanced private tertiary care centers of Pakistan, and caters to a large number of people from all over the country. NICVD is a government-run, tertiary care hospital and as such, is approached by a population more indicative of the country's health status. Pakistan, as a developing country, is home to a vast majority of people living at or below poverty line. Low-cost services at this institute attract a greater number of patients. NICVD was thus chosen for sample selection to increase the reliability of our results and for better generalization of our data to the Pakistani population.

The inclusion criteria were 1) patients of age 18 years and above, 2) those who had been diagnosed with ‘essential’ hypertension and 3) those who were on prescribed antihypertensive medications since at least the previous one month. Patients with co-existing medical conditions were also included. All people who fulfilled the inclusion criteria were then assessed for familiarity with Urdu, which is the national language of Pakistan and is understood and spoken by most people throughout the country, irrespective of ethnicity. Patients who could not converse in Urdu were excluded, due to the lack of appropriate translators.

### Ethical approval, informed consent and patient privacy

Patients who agreed to participate were explained the nature and the objectives of the study, and informed consent was formally obtained. The study was approved by the Ethical Review Committees of AKUH and NICVD. The information about patient's identity was not included with the other data and only the principal investigator had access to this information. No reference to the patient's identity was made at any stage during data analysis or in the paper.

### Data collection

The data collection tool was a questionnaire, designed-based on an extensive literature review of similar studies [23], [24]. The questionnaire was administered by trained interviewers in the Urdu language for ease of comprehension by the patients. The tool was pilot-tested on 50 patients, who were not included in the final study sample. In view of the responses generated in the pilot study, we further modified our survey instrument to include more common responses or to modify the questions. The final survey questionnaire required approximately 20–30 minutes to administer. The questionnaire extracted information regarding patient demographics and clinical characteristics, including co-morbidities such as diabetes, ischemic heart disease, neurological diseases and others, characteristics of hypertension and anti-hypertensive treatment, awareness about hypertension and anti-hypertensive treatment, and factors that, in the patient's views, encouraged or discouraged the patient's drug taking behavior.

Blood pressure was measured twice by the hospital health physicians and trained investigators using aneroid sphygmomanometers and stethoscopes. Blood pressure was measured in all subjects after they were in the resting state for 10 minutes and in sitting position in the right arm place at the level of the heart. An appropriate-sized cuff (cuff bladder encircling at least 80% of the arm) was used to ensure accuracy [2].

Patients were asked about details of their prescribed medication regimen. The information obtained was tabulated. All information was based on self-reporting and included number of prescribed antihypertensive drugs, trade names of all prescribed drugs along with the drug class, doses in milligrams (mg), frequency per day, duration of intake in months, time of intake of the drug (morning, afternoon, evening), and any side effects associated with the drug. A standard drug manual was used to find out the generic class of the drug. Prescriptions, whenever they were available at the time of the interview, were used in getting reliable data, particularly from illiterate patients.

### Depression assessment tool

The Aga Khan University Anxiety and Depression Scale (AKU-ADS) [25] was used to determine the severity of depressive symptoms in the study population. AKU-ADS is an indigenous screening instrument that has been developed in Urdu, for assessment of anxiety-depression syndromes. The questionnaire is based on 25 items, 13 psychological and 12 somatic. At a cut-off score of 20, the sensitivity of the scale is 66%, specificity 79%, positive predictive value 83% and negative predictive value 60%. It covers most of the clinical features specified by DSM-IV criteria, including somatic complaints. Responses to the questions were recorded and scored as never (0), sometimes (1), mostly (2), and always (3). As the scale is designed in Urdu, the chances of differences in interpretation by both the interviewer and the patient were minimal.

### Measurement of adherence

Adherence was defined as ‘the extent to which patients followed their medication schedules as prescribed by their health care providers’ [17]. To measure adherence, patients' self-reports were used. Patients were asked non-judgmentally how often they missed their doses [17]. They were asked the total number of tablets they had been prescribed per week and how many pills they took and missed in the last 3, 5 and 7 days, respectively. Previous studies have adopted assessment over a longer duration of time, sometimes as much as months, to obtain this data. We, however, employed a shorter time period to elicit more accurate responses by minimizing recall bias. Adherence rates were calculated as ‘pills taken over a specific period of time, divided by pills prescribed for that specific period of time’ [17].

To further increase the strength and consistency of our results, we included an adherence assessment through the Morisky Medication Adherence Scale (MMAS) [26], a 4-item questionnaire with a high reliability and validity [14], [26], which has been particularly useful in chronic conditions such as hypertension. It measures both intentional and unintentional adherence based on forgetfulness, carelessness, stopping medication when feeling better, and stopping medication when feeling worse. The scale is scored 1 point for each ‘no’ and 0 points for each ‘yes’. The total score ranges from 0 (non adherent) to 4 (adherent) [27].

### Statistical analyses

All statistical analyses were performed using SPSS 13.0 for Windows. The questionnaire was pre-coded and all data was entered and checked twice by two different investigators. Based on a recent meta-analysis [15] of 129 studies that showed a mean adherence rate of 76.6%, a sample size of 416 participants was calculated for a power of 80% at 5% alpha and with a 6% margin of error. Mean±one standard deviation were computed for all continuous data. Frequencies were calculated for categorical variables. Adherence was represented in percentage and treated as a continuous variable. As described in previous studies, for analysis of adherence a cut-off value of 80% was used for labeling patients as adherent or non-adherent [4], [16], [28], [29]. In univariate analyses, means were compared using student's t-test and ANOVA. Categorical variables were compared using Chi-squared and Fisher's exact tests, as applicable. Odds ratios (with 95% confidence intervals, CI) were calculated from the 2×2 tables. Univariate and multivariate logistic regression were done using adherent *vs.* non-adherent status as the outcome variable, while various study variables were used as independent variables. Associations of study variables with the MMAS score were checked with the help of linear regression using MMAS score as the dependant variable and study variables as independent variables. A p-value of less than 0.05 was considered to be statistically significant for all analyses.

## Results

After pilot-testing and refining the questionnaire with 50 individuals, we interviewed 460 patients at both study centers. After exclusion of cases in which adherence could not be calculated, we included data on 438 cases. In this sample, 71% of the cases were recruited and interviewed at the NICVD, while 29% were recruited from AKUH. There were no statistically significant differences in the adherence and the study variables between the cases recruited from the two hospitals hence combined analysis of the data was done (data not shown). Due to significant difference in prevalence of hypertension in the various ethnic groups in Pakistan, we also recorded the ethnicity as provided by the patient. Ethnicity, however, was not associated with adherence or other study variables and therefore was not analyzed as a risk factor. According to the 80% cutoff level, 77% of the cases were adherent (n = 336, adherence≥80%, mean adherence = 98±5%) and 23% were non-adherent (n = 102, adherence≤80%, mean adherence = 39±29%).

### Demographic and clinical characteristics

There were 199 males (mean age 54±10 years) and 239 females (mean age 50±11 years); 20% of the total cases were younger than 40 and 19% older than 60 years. Although we found adherence to increase with increasing age (*P*<0.02), age remained only marginally different after division of cases into adherent and non-adherent groups. Subjects who were less than 40 years old were less adherent than those older than 70. The highest mean adherence rate was observed in the age group 70–80 years (mean adherence = 91±14%). Although mean adherence showed an overall increase with increasing monthly income and increasing level of education, no significant difference was observed. Table 1 shows the distribution of study variables among the adherent and non-adherent groups. Most of the cases (89%) were married. The mean AKU-ADS score was 19±11 and 190 (43%) cases were depressed according to their AKU-ADS score. A significant proportion of the depressed patients were females 113 (73%, *P*<0.001). Although the presence of a single co-morbid condition slightly increased the adherence, the presence of two or more co-morbids led to a gradual decrease in adherence (*P*>0.05). The most frequently reported co-morbids were diabetes (23.1%) and ischemic heart disease (25.8%). Associations between co-morbidities and adherence, however, were not statistically significant.

**Table 1:** Demographic and clinical characteristics of the study population

	Mean±SD/n (%)	Mean Adherence	Adherent >80%	Non-Adherent <80%	Unadjusted
		n = 438	n = 336	n = 102	OR (95% CI)
Age (years)	52±11	84±29	52±11	49±11	1.0 (1.00–1.04)†
*Gender: n (%)*					
Male	199 (45.4)	83%±31	154 (77.4)	45 (22.6)	NS
Female	239 (54.6)	85%±26	182 (76.2)	57 (23.8)	
*Education status: n (%)*					
Illiterate	120 (27)	81%±33	86 (72)	34 (28)	NS
Primary or below	111 (25)	86%±27	90 (81)	21 (19)	
Intermediate and secondary	124 (28)	84%±28	95 (77)	29 (23)	
Graduate and above	83 (19)	86%±27	65 (78)	18 (22)	
*Monthly Income: (PKR) n (%)*					
Lower (<5000)	173 (39.5)	82%±32	131 (76)	42 (24)	NS
Lower Middle (5k–10k)	120 (27.4)	86%±25	93 (78)	27 (23)	
Middle (10k–20k)	62 (14.2)	80%±32	44 (71)	18 (29)	
Upper Middle (20k–50k)	57 (13)	87%±23	46 (81)	11 (19)	
Upper (>50k)	26 (5.9)	89%±26	22 (85)	4 (15	
*Marital Status: n (%)*					
Single	14 (3.2)	66%±37	7 (50)	7 (50)	NS
Married	379 (86.5)	84%±29	295 (78)	84 (22)	
Divorced**/**Widowed	45 (10.3)	86%±23	34 (76)	11 (24)	
*Depression (AKU-ADS): n (%)*					
Depressed	190 (43.4)	83%±30	142 (75)	48 (25)	NS
Non-depressed	248 (56.6)	85%±28	194 (78)	54 (22)	
*No. of Comorbids: n (%)*					
none	198 (45.2)	83%±29	150 (75.8)	48 (24.2)	NS
1	188 (42.9)	85%±28	147 (78.2)	41 (21.8)	
2	47 (10.7)	84%±27	36 (76.6)	11 (23.4)	
3 or more	5 (1.1)	69%±42	3 (60)	2 (40)	
*Family Hx. of HTN: n (%)*					
Present	212 (48.4)	86%±27	169 (80)	43 (20)	NS
Absent	226 (51.6)	82%±31	167 (74)	59 (26)	

† -P<0.05

HTN–Hypertension, PKR–Pakistani Rupee, AKU–Aga Khan University Hospital, NICVD–National Institute of Cardiovascular Diseases, AKU-ADS-Aga Khan University-Anxiety and Depression Scale, Hx. –History

### Characteristics of hypertension and anti-hypertensive treatment

Table 2 shows the prevalence of various characteristics of hypertension and anti-hypertensive treatment in the adherent and non-adherent groups. Most patients (70.8%) discovered their disease during medical checkup for symptoms related to hypertension and/or its complications.

**Table 2:** Characteristics of hypertension and anti-hypertensive treatment

	Mean±SD/n (%)	Mean Adherence	Adherent >80%	Non-Adherent <80%	Unadjusted
		n = 438	n = 336	n = 102	OR (95% CI)
*Spot BP: (mmHg)*					
Systolic BP	138±23	-	137±22	139±27	NS
Diastolic BP	84±15		83±15	86±17	
*Method of initial diagnosis:*					
Regular checkup	71 (16.2)	87%±28	58 (82)	13 (18)	NS
Checkup for HTN related symptoms	310 (70.8)	84%±28	235 (76)	75 (24)	
Checkup of other causes	57 (13.0)	82%±32	43 (75)	14 (25)	
*HTN related complications:*					
Present	248 (57)	84%±29	193 (78)	55 (22)	NS
Absent	190 (43)	84%±28	143 (75)	47 (25	
*Time since last hospital visit: (months)*					
<24 months	135 (56.5)	88%±25	112 (83)	23 (17)	-
>24 months	104 (43.5)	80%±31	73 (70)	31 (30)	2 (1.1–3.8)†
*No. of Antihypertensive drugs:*					
Monotherapy	201 (46)	79%±31	138 (69)	63 (31)	0.3 (0.1–0.6)†
Two drugs	163 (37)	87%±27	133 (82)	30 (18)	0.6 (0.3–1.4)
Three drugs or more	74 (17)	90%±24	65 (87)	9 (12)	-
*Who pays for medication:*					
Self	166 (38)	82%±31	124 (75)	42 (25)	NS
Family	162 (37)	85%±27	127 (78)	35 (22)	
Welfare/Charity	80 (18)	81%±30	59 (74)	21 (26)	
Employer	29 (6.6)	93%±17	25 (86)	4 (14)	

† -P<0.05

HTN–Hypertension, BP–Blood pressure.

A greater proportion of the cases suffering from hypertension-related complications were adherent (*P*>0.05). A large proportion of our study sample had never been hospitalized (45%). The patients who had been hospitalized in the previous two years had significantly higher adherence (*P*<0.05). Surprisingly adherence increased with increasing number of anti-hypertensive drugs that a person was taking (*P*<0.05).

### Awareness about hypertension and anti-hypertensive treatment

The overall level of awareness about hypertension and its treatment was very low. As shown in Table 3, 24% of the study sample took their medication only when they thought they had symptoms of high blood pressure. This patient group had very low adherence. The patients who considered every dose to affect blood pressure had significantly higher adherence (*P*<0.001) and lower systolic and diastolic blood pressures. A very small proportion of patients were aware of the risk factors for hypertension and an even smaller proportion knew about the complications. Greater awareness was associated with higher adherence.

**Table 3:** Awareness about hypertension and anti-hypertensive treatment

	Mean±SD/n (%)	Mean Adherence	Adherent	Non-Adherent	Unadjusted
		n = 438	Adherence >80%	Adherence <80%	OR (95% CI)
			n = 336	n = 102	
*When do you take your medication:*					
Regularly	332 (76)	93%±16	291 (88)	41 (12)	-
For symptomatic relief	106 (24)	57%±40	45 (43)	61 (58)	9.6 (5.8–15.9)†
*In your view your blood pressure is:*					
Controlled	313 (72)	86%±26	245 (78)	68 (22)	NS
Uncontrolled	124 (28)	79%±33	91 (73)	33 (27)	
*Missing a single dose:*					
Does not affect BP	192 (44)	77%±33	131 (68)	61 (32)	2.3 (1.5–3.7)†
Affects BP	246 (56)	90%±23	205 (83)	41 (17)	-
*Following increases risk for HTN:*					
Smoking	51 (12)		39 (77)	12 (24)	NS
Alcohol	9 (2)		9 (100)	−49	NS
High salt	165 (38)		116 (70)	(30)	0.6 (0.4–0.9)†
Diabetes Mellitus	26 (5.9)		24 (92)	2 (8)	NS
Obesity	44 (10)	-	37 (84)	7 (16)	NS
Male gender	18 (4.1)		15 (83)	3 (17)	NS
Stress	276 (63)		202 (73)	74 (27)	0.6 (0.4–0.9)†
Age	31 (7)		27 (87)	4 (13)	NS
Family History	61 (14)		54 (89)	7 (12)	2.6 (1.1–5.9)†
Physical inactivity	32 (7.3)		27 (84)	5 (16)	NS
*Following organs are affected by HTN:*					
Heart	297 (68)		226 (76)	71 (24)	NS
CNS	148 (34)	-	114 (77)	34 (23)	NS
Eyes	45 (10)		36 (80)	9 (20)	NS
Kidney	95 (22)		81 (85)	14 (15)	2 (1.0–3.7)†
Vasculature	34 (8)		24 (71)	10 (29)	NS
Extra Salt added to diet	33 (7.5)	83%±31	25 (76)	8 (24)	NS

† -P<0.05

HTN–Hypertension, BP–Blood pressure.

### Factors associated with low MMAS

The mean MMAS score in the overall sample was 2.5±1.3. MMAS score was significantly higher (*P*<0.001) in the adherent group (2.7±1.2) compared to the non-adherent group (1.7±1). Table 4 lists the patient variables significantly associated with the MMAS. MMAS scores of ≤2 were associated with a mean adherence <73%. The patients with MMAS scores ≤2 were relatively younger (51 *vs.* 53 years), were illiterate or at a lower level of education (*P*<0.05), belonged to a lower income subgroup (*P*<0.05), and had higher systolic (140±26 *vs.* 136±19 mmHg, *P*<0.05) and diastolic (86±18 *vs.* 82±13, *P*<0.01) pressures. A significantly greater proportion of patients with MMAS score ≤2 were depressed (50% *vs.* 38%, *P*<0.05), according to AKU-ADS.

**Table 4:** Patient factors associated with MMAS

	β (95% CI)	p value
Adherence	0.016 (0.012–0.02)	<0.001
Age	0.01 (0.002–0.03)	0.02
Education status	0.12 (0.01–0.2)	0.03
Monthly Income	0.16 (0.06–0.26)	0.001
No. of prescribed drugs	0.17 (0.08–0.33)	0.04
Patient's assessment of his/her blood pressure	−0.36 (−0.63– −0.1)	0.007
Does missing a dose make any difference?	−0.58 (−0.82– −0.34)	<0.001
Do you take you medication regularly or only for symptomatic relief?	−1.2 (−1.5– −0.98)	<0.001
Presence of depression	0.34 (0.1–0.6)	0.005

† -P<0.05

Table 5 shows the prevalence of various personal, social and behavioral characteristics that in the patient's views affected their drug adherence. The prevalence of most of the factors was significantly different in the adherent and non-adherent groups. While the common encouraging factors, such as understanding the need and effectiveness of the prescribed medication and availability of support system, were significantly associated with better adherence, the most common discouraging factors cited in literature such as forgetfulness [22], side effects [16], [17], cost of medication [16] and lack of access to medication [14] did not show statistically significant associations with non-adherence.

**Table 5:** Factors that, in the patient’s views, encouraged or discouraged the patient’s drug taking behavior

Common Encouraging Factors	n (%)	Adherent	Non-Adherent	p value
		Adherence >80%	Adherence <80%	
		n = 336	n = 102	
Understanding the need of medication	360 (82)	296 (82)	64 (18)	<0.001
Understanding the effectiveness of medication	378 (86)	303 (80)	75 (20)	<0.001
Availability of Support System	235 (54)	192 (82)	43 (18)	0.008
Making medication a habit	244 (56)	206 (84)	38 (16)	<0.001
Ready Availability of medication	266 (61)	214 (81)	52 (19)	NS
Good relationship with the doctor	262 (60)	209 (80)	53 (20)	NS
Keeping medication in sight	177 (40)	153 (86)	24 (14)	<0.001
**Common Discouraging Factors**				
Lack of understanding of need of medication	40 (9)	12 (30)	28 (70)	<0.001
Lack of understanding of effectiveness of medication	32 (7)	13 (41)	19 (59)	<0.001
Lack of support system	44 (10)	22 (50)	22 (50)	<0.001
Lack of access to medication	54 (12)	34 (63)	20 (37)	0.01
Fear of getting used to medication	117 (27)	73 (62)	44 (38)	<0.001
Cost of medication	177 (40)	126 (71)	51 (29)	0.02
Forgetting	210 (48)	150 (71)	60 (29)	0.01
Side effects	73 (17)	47 (64)	26 (36)	0.006
Dissatisfaction with treatment	56 (13)	31 (55)	25 (45)	<0.001
Traveling	115 (26)	75 (65)	40 (35)	0.001

† -P<0.05

Upon multivariate analysis, the only factors associated with adherence were the number of drugs that a patient was taking (*P*<0.02) and whether he/she was taking medication regularly or only for symptomatic relief (*P*<0.00001)

## Discussion

Adherence to antihypertensive therapy as measured by our study was 77% in the studied Pakistani population, when defined by the ≥80% cut off. The factors showing significant associations with adherence were age, number of drugs prescribed and patients' knowledge of the disease and treatment, including their beliefs and practices.

Our study reports a higher adherence in the Pakistani population than what has been reported previously in a local study (57%) [22]. This could be due to measurement of adherence based on different criteria in the two studies, along with variation in the subset of population which served as the study sample.

Among the studies conducted on various populations of the world, using a similar cut-off, the adherence we observe is higher than what has been reported in a similar study in Malaysia (44.2%) [16], comparable to a study in Egypt (74.1%) [23] and lower than what a study in the Western population (Scotland) reports (91%) [4]. Hence, we found that people of a developing country, like Pakistan, are generally more adherent to their medication than what might be assumed. However, population studies with larger samples are needed to support our claim.

Age was found to be significantly and independently associated with adherence in our study, with better adherence observed in older people. This finding is consistent with a number of other studies [30], [31], [32] including the regional study in Malaysia [16], although there are studies which show either no association [4], [23] or decreasing adherence with increasing age [33]. Increasing self-reliance in old age has been shown to decrease adherence [34]. In the Pakistani population, a better social support structure ensured by the common extended family system, reduces self-reliance and could be the reason for better adherence in this age group. It is usual for other family members to take full responsibility of the medication routine of the families' patients.

An inverse relationship was observed between adherence and number of pills prescribed. Patients on monotherapy had a mean adherence of 79% compared to 90% for those on three drugs or more (OR; 95% CI, 0.3; 0.1–0.6). This is in contrast to what has been frequently reported so far. A recent meta-analysis of eight studies reports that the average adherence for once-daily dosing was significantly higher than for multiple daily dosing (91.4% *vs.* 83.2%, respectively, *P*<0.001) [35]. Some latest studies, however, have identified no relation between increasing number of drugs and poor adherence [4], including one such study in an Asian population [16]. One reason for our finding could be that patients on multiple pills feel that the severity of their disease is significant and hence become more cautious with their treatment, compared to those on monotherapy, who may take treatment lightly. Another reason may perhaps be that when patients have to take multiple medications, they are less likely to forget to take them, compared to having to take only one pill.

Baune *et al,* showed a significant correlation between education and QOL among patients with hypertension in Gaza Strip and hypothesized that educational interventions would be essential in preventing high blood pressures and consequent mortality [36]. Knowledge of hypertension significantly affected adherence in our study sample. Patients who were aware of the association between certain risk factors for hypertension, such as high salt intake, stress and a positive family history, had better adherence compared to those who with poorer knowledge. Studies from the developed world, however, indicate no association between patients' knowledge and adherence [32], [37].

Patients' beliefs and attitudes have been explored in studies worldwide to explain not taking medication as prescribed [17]. Egan *et al*. found forgetfulness, adverse effects and not liking to take medication among the reasons for poor adherence in a nationally representative sample in the United States [38]. Commonly encouraging factors, such as understanding the need and effectiveness of medication, a good support system and employing methods to reduce forgetfulness such as keeping medication in sight, were all significantly associated with better adherence in our population. Similarly, among the discouraging factors cited in literature, most commonly reported in our population were forgetfulness (48%) as has been reported by an earlier local study [22], followed by cost (40%) and fear of getting used to medication (27%). These were, however, factors that reduced adherence among the adherent (>80% adherence) population. This was different from the major factors reducing adherence in the non-adherent (<80% adherence) patients, whose main issues were lack of understanding of need of medication (70%) and lack of understanding of effectiveness of medication (59%).

Depression has recently been added to the list of factors associated with non-adherence to anti-hypertensive medication [32]. Wang *et al*. [21] demonstrated a significant association between depression, as a multivariate factor, and non-adherence. In our study, however, depression was not found to independently correlate with non-adherence.

Our study design was limited in several aspects. Self-reporting was used as the only method of measuring adherence. Although this method has the disadvantages of recall bias, of eliciting only socially acceptable responses and hence, may overestimate adherence [14], it is simple, economically feasible and the most useful method in clinical settings [17]. Response rate of the participants was not recorded and thus in this study it is assumed that responders and non-responders are similar in distribution of the recorded variables. Apart from harboring the known limitations of the cross-sectional design, our study involved patients recruited from tertiary care hospitals only, and hence the results cannot be generalized. As we included patients with co-existing illnesses, some of our results may not be purely indicative of the characteristics of hypertensive patients. We did not include patients who could not converse in Urdu and this further restricts the generalization of our findings. Most adherence studies based on self-reporting ask patients to give information about long durations (usually ranging from 1 month to 1 year) to avoid any bias introduced by the brevity of the duration. Increasing the duration of time period could give a more generalized view of the patient's adherence over a longer period of time but at the same time increases the chance of introducing recall bias. Self-reported adherence based on a short duration of time has equal chances of being under-reported as over-reported, depending on the patient's behavior in the recent past, but minimizes the chance of recall bias and hence is more accurate. The published literature suggests that people are more accurate in reporting non-adherence when asked simple questions about recent behavior [27], [39].

The adherence goal of 80% of prescribed dose is used conventionally in clinical trials of safety and efficacy [4]. Hence, we used this value as a cut point for labeling patients as adherent and non-adherent. All the analyses, however, were repeated using a higher cut-off of 90% in order to dichotomize adherence and the results reported here were found to be consistent.

The use of validated tools in our study further strengthens the reliability of our results. MMAS had a significant linear relation with adherence measured as a continuum. Most of the factors associated with non-adherence discussed above were associated with low MMAS scores of ≤2, including those which did not show an association with adherence at the cut-off of 80%, such as depression. More studies, however, are needed to demonstrate the validity of MMAS in the Pakistani population. Hence, we have reported only those factors as significantly affecting adherence that were common to both our criteria for measurement of adherence.

In conclusion, we found younger age, monotherapy, poor awareness and symptomatic treatment to be the strongest factors affecting adherence to anti-hypertensive medication amongst Pakistani patients. Future studies are recommended to confirm our findings, as adherence to medication predicts better outcomes and indicators of poor adherence to a medication regimen are a useful resource for physicians to help identify patients who are most in need of interventions to improve adherence.

We recommend the implementation of education campaigns to increase awareness about the risk factors, natural history, complications and treatment of hypertension. Global events, such as World Hypertension Day, could be used as a forum to highlight these issues. Patient support groups can be employed to help the non-adherent. Patients who have suffered complications due to non-adherence could be requested to voluntarily share their experiences. Print and audiovisual media would be very helpful in dissemination of information. Most importantly, though, physicians have to pay special attention to patient education and counseling when treating hypertensive patients.
